# Heterologous expression of *Oenococcus oeni *malolactic enzyme in *Lactobacillus plantarum *for improved malolactic fermentation

**DOI:** 10.1186/2191-0855-2-19

**Published:** 2012-03-27

**Authors:** Christina Schümann, Herbert Michlmayr, Reinhard Eder, Andrés M del Hierro, Klaus D Kulbe, Geir Mathiesen, Thu-Ha Nguyen

**Affiliations:** 1 Food Biotechnology Lab, Department of Food Sciences and Technology, University of Natural Resources and Life Sciences, Vienna, Austria; 2 Federal College and Research Institute for Viticulture and Pomology (HBLAuBA), Klosterneuburg, Austria; 3 Department of Chemistry, Biotechnology and Food Science, Norwegian University of Life Sciences, Ås, Norway

**Keywords:** *L. plantarum*, *Oenococcus oeni*, Malolactic fermentation, Malolactic enzyme

## Abstract

*Lactobacillus plantarum *is involved in a multitude of food related industrial fermentation processes including the malolactic fermentation (MLF) of wine. This work is the first report on a recombinant *L. plantarum *strain successfully conducting MLF. The malolactic enzyme (MLE) from *Oenococcus oeni *was cloned into the lactobacillal expression vector pSIP409 which is based on the sakacin P operon of *Lactobacillus sakei *and expressed in the host strain *L. plantarum *WCFS1. Both recombinant and wild-type *L. plantarum *strains were tested for MLF using a buffered malic acid solution in absence of glucose. Under the conditions with L-malic acid as the only energy source and in presence of Mn^2+ ^and NAD^+^, the recombinant *L. plantarum *and the wild-type strain converted 85% (2.5 g/l) and 51% (1.5 g/l), respectively, of L-malic acid in 3.5 days. Furthermore, the recombinant *L. plantarum *cells converted in a modified wine 15% (0.4 g/l) of initial L-malic acid concentration in 2 days. In conclusion, recombinant *L. plantarum *cells expressing MLE accelerate the malolactic fermentation.

## Introduction

Lactic acid bacteria (LAB) contribute to taste and texture of a wide range of fermented products and inhibit the growth of spoilage bacteria (Mozzi et al. 2010). This includes the application of LAB in winemaking to increase the stability of wines that undergo barrel or bottle-ageing. This process, the malolactic fermentation (MLF), normally occurs after the alcoholic fermentation (AF). Apart from the decarboxylation of L-malic into L-lactic acid, MLF removes carbon sources of other microorganisms and bestows sensory changes to the wine (Bartowsky 2005). The genera mainly found during MLF are *Lactobacillus, Leuconostoc, Pediococcus *and *Oenococcus*. Due to its high tolerance to low pH and higher amounts of SO_2 _and ethanol, *Oenococcos oeni *is the primary species encountered during spontaneous MLF (Capozzi et al. 2010). Furthermore, *O. oeni *is the preferred organism for malolactic starter cultures, since the presence of *Lactobacillus *sp. and *Pediococcus *sp. during MLF can lead to development of spoilage aroma and off-flavours (Moreno-Arribas and Polo 2005). However, also *O. oeni *is able to generate for example acetic acid, diacetyl (buttery flavour), mannitol (viscous, sweet) and mousy off-flavour (Bartowsky 2009). Additionally, *O. oeni *needs several weeks to degrade malic acid completely, and growth of LAB is often delayed or can even fail (Zhang and Lovitt 2005). Consequently, innovations are desirable to reduce malic acid in a faster and more efficient way.

The enzymatic nature of the MLF was first observed in a crude extract of *L. plantarum *(formerly called *L. arabinosus*) in 1948 (Korkes and Ochoa 1948). Initially, it was presumed that the decarboxylation of malic acid originates from an enzyme cascade, until Caspritz and Radler (Caspritz and Radler 1983) proofed that a single enzyme directly converts L-malic to L-lactic acid. This enzyme, usually referred to as the malolactic enzyme (MLE, not EC classified), is only active in the presence of catalytical concentrations of NAD^+ ^and Mn^2+^. To date the mechanism of the MLE is still unclear.

The pH optimum of the MLE is around pH 6.0, therefore direct application to must or wine, where the pH is below 4.0, is not possible (Costantini et al. 2009). Several attempts to immobilize bacteria or even the MLE have been performed in order to improve the control and the yield of the MLF (Maicas 2001). The sequencing of the *mle *gene from *Lactococcus lactis *(Ansanay et al. 1993) followed by the description of the complete mle operon from *O. oeni *(Labarre et al. 1996) opened up new possibilities for genetic modifications aiming a more efficient MLF. Consequently, the MLE has been expressed in several organisms. An overview, including the conversion of L-malic acid per day by these strains, is shown in Table 1. One of the approaches is the use of *Saccharomyces cerevisiae*, allowing simultaneous alcoholic - and malolactic fermentation. Two accordingly modified yeast strains are already on the market in some countries (Sablayrolles 2009). However, this approach affects the flavour profile of the final wine resulting for example in less metabolised ethyl lactate and subsequent decreased mouthfeel (Husnik et al. 2007). Winemakers are dependent on the aroma given by LAB to create specific styles which is forced by consumer preferences for new product development (Lerm et al. 2010). For that reason, an alternative approach might be the application of genetically modified LAB, heterologously expressing the MLE, to perform MLF in a shorter time and to achieve new and possibly more attractive flavour variations.

**Table 1:** Overview of recent work of MLE production in recombinant *Escherichia coli, Lactobacillus plantarum, Saccharomyces cerevisiae *and *Schizosaccharomyces pombe*.

Source of *mle*	Expression host	L-malic acid degradation (g/l per day)	Specific activity of crude enzyme (U/mg)	References
*Lactobacillus delbrueckii*	*Escherichia coli*	0.05	ND	Williams et al. (1984)

	*Saccharomyces cerevisiae*	0.01	ND	
*Lactococcus lactis*	*Escherichia coli*	0.23	ND	Ansanay et al. (1993)
	*Saccharomyces cerevisiae*	0.39 (pH 3.0)	ND	
	*Escherichia coli*	ND	0.27	Denayrolles et al. (1994)
	*Saccharomyces cerevisiae*	0.08 (pH 3.0)	0.7	Denayrolles et al. (1995)
	*Saccharomyces cerevisiae*	0.14 (pH 3.5)	18	Ansanay et al. (1996)
	*Schizosaccharomyces pombe*	1.50*,^+ ^(pH 3.5)	ND	
	*Saccharomyces cerevisiae*	0.72^+ ^(pH 3.5)	ND	Bony et al. (1997)

*Pediococcus damnosus*	*Saccharomyces cerevisiae*	2.00^+ ^(pH 3.3)	ND	Bauer et al. (2005)

	*Escherichia coli*	Not detectable	0.13**	Labarre et al. (1996)

*Oenococcus oeni*	*Saccharomyces cerevisiae*	1,05 (pH 3.0)	0.02	Labarre et al. (1996)
	*Saccharomyces cerevisiae*	1.81^+ ^(pH 3.5)	ND	Husnik et al. (2007)
	*Saccharomyces cerevisiae*	0.34	ND	Liu and Li (2009)
	*Escherichia coli*	ND	14.9	Schümann (personal communication))
	*Lactobacillus plantarum*	5.0*,^+ ^(pH 4.0)	22.1	This work

Summary of available data on the consumption of L-malic acid (g/l) per day from medium, pH is indicated in parenthesis. The specific activities of the crude extracts (CE) are presented in μmol/ml per min and mg protein (U/mg). *Schizosaccharomyces pombe*

ND, not determined

*conversion of malic acid similar to the control

**after ammonium sulfate precipitation

^+^complete conversion of malic acid

Several inducible and controlled expressions systems have been developed for LAB. One of the best known systems is perhaps the nisin-controlled gene expression system (NICE) for *L. lactis *(Mierau and Kleerebezem 2005). However, *L. lactis *is not a wine related LAB. On the other hand, *L. plantarum *occurs at different stages of wine production and performs MLF (Sáenz et al. 2009, Todorov and de Melo Franco 2010). An expression system based on the genes involved in sakacin P production has been developed for use in *L. plantarum *and is successfully applied for the production of different proteins (Sørvig et al. 2003, Sørvig et al. 2005, Halbmayr et al. 2008, Kolandaswamy et al. 2009). In the present study, we demonstrated the use of this expression system to clone and express the *mle *gene from *O. oeni *into *L. plantarum *and utilize the recombinant *L. plantarum *for malolactic fermentation.

## Materials and methods

### Chemicals and enzymes

Unless otherwise stated, all chemicals were obtained from Sigma-Aldrich (Steinheim, Germany) or Roth (Karlsruhe, Germany) and were of reagent grade. All restriction enzymes, T4 DNA ligase and Phusion High-Fidelity PCR Master Mix (Finnzymes) were obtained from New England Biolabs (NEB, Frankfurt, Germany) while REDTaq ReadyMix PCR Reaction Mix was purchased from Sigma-Aldrich.

### Bacterial strains and growth conditions

The organisms used in this study, *O. oeni *DSM 20252 and DSM 20255, purchased from the German Collection of Microorganisms and Cell Cultures (DSMZ, Braunschweig, Germany), *E. coli *OneShot TOP10 cells were from Invitrogen (Carlsbad, CA, USA). *L. plantarum *WCFS1, a single colony isolate of strain NCIMB8826 that was originally isolated from human saliva (Kleerebezem et al. 2003), is from the culture collection of the Norwegian University of Life Sciences, Ås, Norway. *O. oeni *(25°C) and *L. plantarum *(30°C) were grown in de Man-Rogosa-Sharp (MRS) broth (de Man et al. 1960) and when appropriate supplemented with erythromycin (5 μg/ml). *E. coli *was grown at 37°C in Luria-Bertani (LB) medium (Bertani 1951) with addition of 200 μg/ml erythromycin, when necessary. Agar plates were either made of MRS-agar (Merck, Darmstadt, Germany) or LB media including 15 g/l agar.

### DNA isolation and sequence analysis

Polymerase chain reaction (PCR) amplifications, restriction enzyme digestion, agarose gel electrophoresis, plasmid DNA isolation, and transformation in *E. coli *were performed as described previously (Sambrook and Russell 2001). The genes encoding the MLE from *O. oeni *DSM 20252 and 20255 were amplified from chromosomal DNA and the sequences are deposited in the GenBank database with the accession numbers GQ911572 and GQ924754, respectively.

### Construction of expression vector

For heterologous expression of the MLE in *L. plantarum *the sakacin P based expression system (pSIP-vectors) (Sørvig et al. 2003) was used. The *mle *gene from *O. oeni *20255 was amplified from genomic DNA using primers GATGATCTCGAGAAAAGA**CATCATCATCATCATCAT**GGTGGAGACTACAAG*GATGACGATGACAAG*ATGACAGATCCAGTAAGTATTTTA and GAGCTCGAATTCTTAGTATTTCGGATCCCAC to introduce a N-terminal tag consisting of an His_6_-tag (bold) and the enteropeptidase (enterokinase, EC 3.4.21.9) restriction site (italic). Subsequently, the PCR product and the vector pSIP409 were digested by restriction enzymes *Xho*I and *EcoR*I (underlined). Both fragments were ligated and the resulting plasmid was transformed into electrocompetent *L. plantarum *cells as described previously (Aukrust, and Blom 1992). Positive colony PCR amplified constructs were verified by sequencing, performed by a commercial provider, and the plasmid was named pSC9mle.

### Expression and purification of recombinant enzyme

The recombinant *L. plantarum *harbouring pSC9mle was cultivated in 0.5 litre MRS broth, inoculated from a 10 ml overnight culture. Thereafter, cells were grown for 8 h at 30°C, before induction with 25 ng/ml peptide pheromone IP-673. After an induction time of 14 h at 25°C the cells were harvested by centrifugation (4000 × g, 10 min, 4°C), washed three times with 0.9% NaCl solution and resuspended in wash buffer (100 mM N-2-hydroxyethylpiperazine-N'-2-ethanesulfonic acid (HEPES), 100 mM KCl, 20 mM imidazole (AppliChem, Darmstadt, Germany), pH 6.0. The harvested cells were disrupted by using a French press (Aminco, Maryland, USA) and the cell debris was removed by ultracentrifugation (100,000 × g, 30 min, 4°C). The MLE was purified using immobilized metal ion affinity chromatography (IMAC) column (15 ml - Bio-Rad Laboratories, Hercules, CA) that was equilibrated with wash buffer. The protein was eluted at a rate of 2 ml/min with elution buffer containing 1 M imidazole. Active fractions were pooled, desalted, concentrated and stored in 100 mM HEPES, 0.5 mM NAD^+ ^and 0.1 mM Mn^2+ ^(pH 6.0).

### Activity assay and determination of protein and molecular mass

Activity of the MLE was determined by measuring the decreasing amount of malic acid and increasing amount of lactic acid in the assay. The reaction mixture contained 100 mM HEPES (pH 6.0), 0.5 mM NAD^+^, 0.1 mM Mn^2+ ^and 15 mM L-malic acid (pH 6.0), and was incubated at 45°C using an Eppendorf thermomixer. The reaction was started with the addition of 20 μl enzyme and stopped after 5 min reaction time by heating at 70°C for 1 min to inactivate the enzyme.

The influence of pH and temperature on the activity of the recombinant malolactic enzyme was studied. HEPES buffer and L-malic acid solution were adjusted to pH between 5.0 and 7.0 with KOH and the analysis were performed as described above in the temperature range from 30°C to 50°C.

Organic acids were analyzed by high performance liquid chromatography (HPLC) using a Dionex System (Summit and Chromeleon software, Sunnyvale, CA, USA) equipped with a Supelcogel H column (25 cm × 4.6 mm) from Sigma-Aldrich (40°C, 0.1% H_3_PO_4_, 0.2 ml min^-1^, injection volume: 20 μl) and a 210 nm UV detector. To confirm specificity to the L- form of malic and lactic acid, both acids were further quantified with enzymatic test kits from Roche, purchased from R-Biopharm (Darmstadt, Germany). The enzyme activity (U) is expressed as μmol of L-malic acid converted per minute at 45°C.

The protein concentration was determined using the method of (Bradford 1976) with bovine serum albumin as standard. Protein samples were analyzed by sodium dodecyl sulfate polyacrylamide gel electrophoresis (SDS-PAGE) (Laemmli 1970). Coomassie blue staining was used for the visualization of the protein bands.

### Influence of the pH in MRS medium on the heterologous MLE production

The influence of pH on the plasmid stability and on the subsequent production of MLE was determined in MRS medium containing 5 μg/ml erythromycin. The recombinant strain was first grown in 5 ml MRS medium at initial pH of 4.0, 5.0 and 6.0. The resulting cells were diluted into 100 ml MRS medium in appropriate pH to final OD_600 _of 0.05 and further incubated until OD_600 _reached at least 0.2 before induction with 25 ng/ml peptide pheromone IP-673. After 24 h, including 19 h of induction time at 25°C, the induced cells were harvested (4.000 rpm, 10 min, 4°C), washed twice with 0.9% NaCl solution and resuspended in HEPES buffer (pH 6.0). Thereafter, 0.5 ml cell suspension was homogenized in Precellyse 24 (Bertin Technology, Montigny, France) in presence of 0.5 g glass beads (0.5 mm) and after centrifugation (16.000 rpm, 10 min, 4°C) MLE activity in the cell free supernatant was determined.

The wild-type and the recombinant *L. plantarum *strains were also tested for their efficiency to decarboxylate L-malic acid in a medium at pH 4.0. These strains were cultivated in 250 ml MRS-medium including 5 g/l L-malic acid at 25°C for 24 h with initial OD_600 _of 0.1 and the recombinant strain was induced for 12 h. Samples, taken at regular intervals, were assayed for cell density (OD_600_) and quantity of L-malic acid. All experiments were performed in triplicate.

### Reduction of acidity in a malic acid solution

Cells of the wild-type and the recombinant *L. plantarum*, harbouring pSC9mle, were used to compare their efficiency to convert L-malic acid. Both strains were cultivated anaerobically in 300 ml MRS broth containing 5 g/l L-malic acid. Additionally, the recombinant strain was also cultivated in the absence of L-malic acid. Cultures with initial cell density of 0.2 (OD_600_) were grown at 30°C for 2 h before the recombinant strains were induced with 25 ng/ml peptide pheromone. Cells were harvested (4,000 rpm, 10 min, 20°C) after 6 h of induction at 25°C, washed once with 500 ml 0.9% NaCl solution and resuspended in malolactic test solution (3 g/l L-malic acid, pH 5.0). Intracellular MLE activity was determined as described above. The cell suspensions were further diluted to a final OD_600 _of approximately 0.1 in 200 ml malolactic test solution and 200 ml malolactic test solution was supplemented with 0.1 mM NAD^+ ^and 0.02 mM Mn^2+^. The malolactic test solution contained no antibiotics and inducing agent due to the use of pre-induced cells. These solutions were incubated at 25°C with regular sampling to measure the cell density (OD_600_) and the content of malic and lactic acid by HPLC as described above. The according number of colony forming units (CFU) was determined using the most probable number (MPN) method. Therefore three serials of 10-fold dilutions were prepared in MRS medium, starting with 1 ml sample of wild-type *L. plantarum *cells (from inoculated malolactic test solution) and incubated at 30°C for 48 h.

### Decarboxylation of malic acid in modified wine

The wild-type strain and the recombinant *L. plantarum *strain were also applied for conducting MLF in modified wine. The wine used, a Grüner Veltliner from vintage 2010, was adjusted to pH 5.0 by deacidification (original total acid of 8 g/L and 3 g/L malic acid) with CaCO_3_. The deacidified wine had a final chemical analysis of 11.8 v/v alcohol, 24 mg/l free SO_2_, 76 mg/l total SO_2_, 0.2 g/l glucose, 0.4 g/l fructose and 2.3 g/l L-malic acid with a pH of 4.8. This wine was further manipulated by addition of L-malic acid and adjustment to pH 5.0 with 4.0 M KOH. To avoid possibilities of contamination the final wine was sterile filtered (0.22 μm filter membrane). Cells used for the experiment with modified wine, containing additionally Mn^2+ ^and NAD^+^, were treated the same as described for the application in malolactic test solution.

The behaviour of cells, which are being able to adapt to wine, were investigated. Both strains were pre-cultured as described above with the following modifications: the pre-cultures were grown in 100 ml MRS medium containing 5 g/l L-malic acid over night and were used to inoculate 100 ml MRS medium diluted with 25% modified wine (pH 5.7). After 12 h incubation 1 ml of these cultures, containing the cells that were pre-adapted to MRS medium with 25% modified wine, were inoculated to 100 ml medium containing 50% modified wine (pH 5.6) and inducted when the cell density reached ~0.2 (OD_600_). The cells were harvested after 12 h induction. Each experiment was performed in triplicate in 250 ml modified wine at 25°C. Samples were taken regularly and tested spectrophotometrically for cell density (OD_600_) and enzymatically for the content of L-malic acid as described above.

## Results

### Cloning, production and purification of the MLE in *L. plantarum*

**Figure 1:**
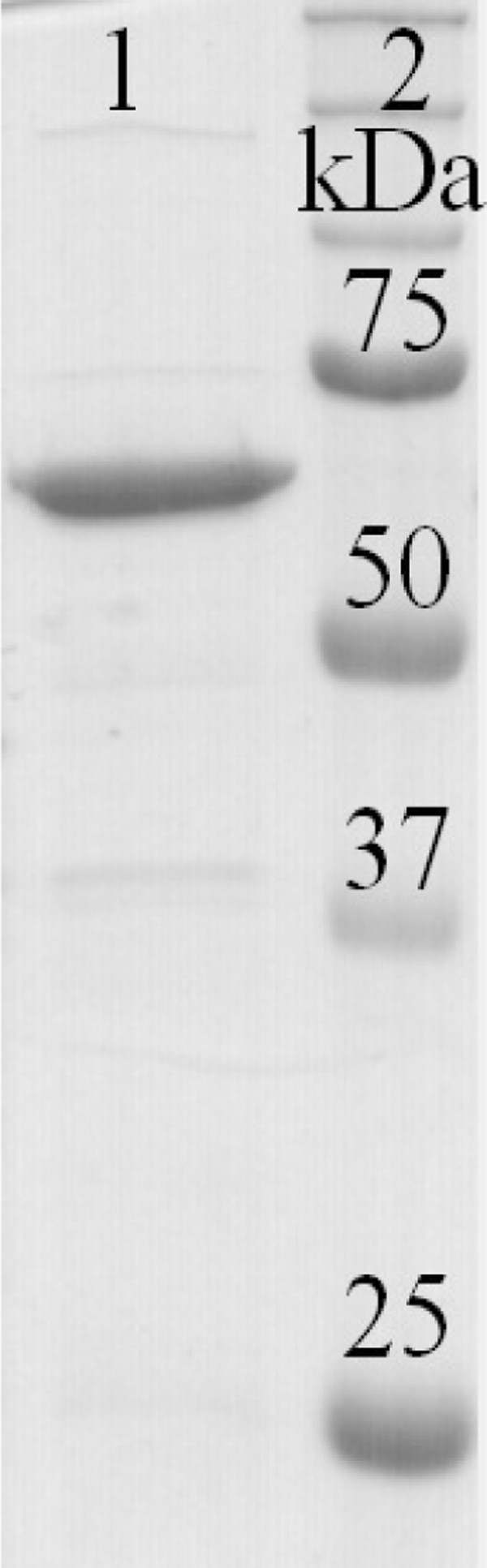
**Coomassie Blue stained gel after SDS-PAGE with purified MLE produced in *L. plantarum *(lane 1) and molecular mass marker (Bio-Rad) (lane 2)**.

The *mle *gene from *O. oeni *20255 (accession No. GQ924754) was cloned into pSIP409 vector (Sørvig et al. 2005) to express the MLE in *L. plantarum*. The resulting plasmid pSC9mle encoded the following N-terminal leader sequence MVACSSRLEKRHHHHHHGGDYKDDDDKX including a His_6 _affinity tag in frame fused to the *mle *gene. Sequence analysis confirmed the *mle *gene encodes a protein of 568 amino acids with a calculated molecular mass of 62.3 kDa and after purification a single band with a molecular mass of ~60 kDa appeared on the SDS-PAGE gel (Figure 1). The expression resulted in approximately 6.6 kU of the recombinant MLE per litre fermentation broth with a specific activity of 22.1 U/mg (Table 2). After purification using profinity IMAC resins the specific activity increased approximately 10 folds to 217 U/mg with a recovery of 53%.

**Table 2:** Purification of the recombinant MLE.

Purification step	Total activity (U)	Total protein (mg)	Specific activity (U/mg)	Purification (fold)	Recovery (%)
Crude extract	3,289	148.7	22.1	1.0	100
Affinity chromatography	1,748	8.05	217	9.8	53

The MLE was expressed in 0.5 litre and values are the mean of duplicate experiments

### Influence of pH and temperature on the purified recombinant enzyme

**Figure 2:**
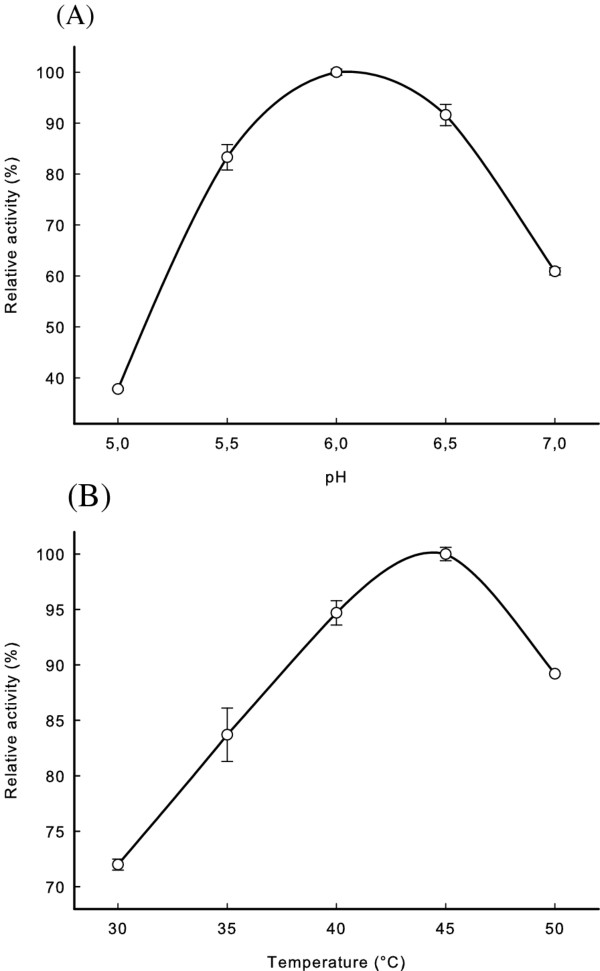
**Relative activity of *O. oeni *MLE at different pH (A) or temperature (B)**. Values are the average of duplicate experiments with standard deviation shown as error bars.

The pH optimum of the purified enzyme was tested in HEPES buffer. The activity was highest between pH 5.5 and 6.5, with maximum activity at pH 6.0 (Figure 2A). The temperature optimum was determined to be 45°C when using HEPES buffer at pH 6.0 (Figure 2B). The enzyme activity decreased significantly above and below this temperature, and at 30°C the MLE retained about 72% of the activity compared to the activity at 45°C (Figure 2B).

### Effect of the pH in the growth medium on the production of MLE

**Figure 3:**
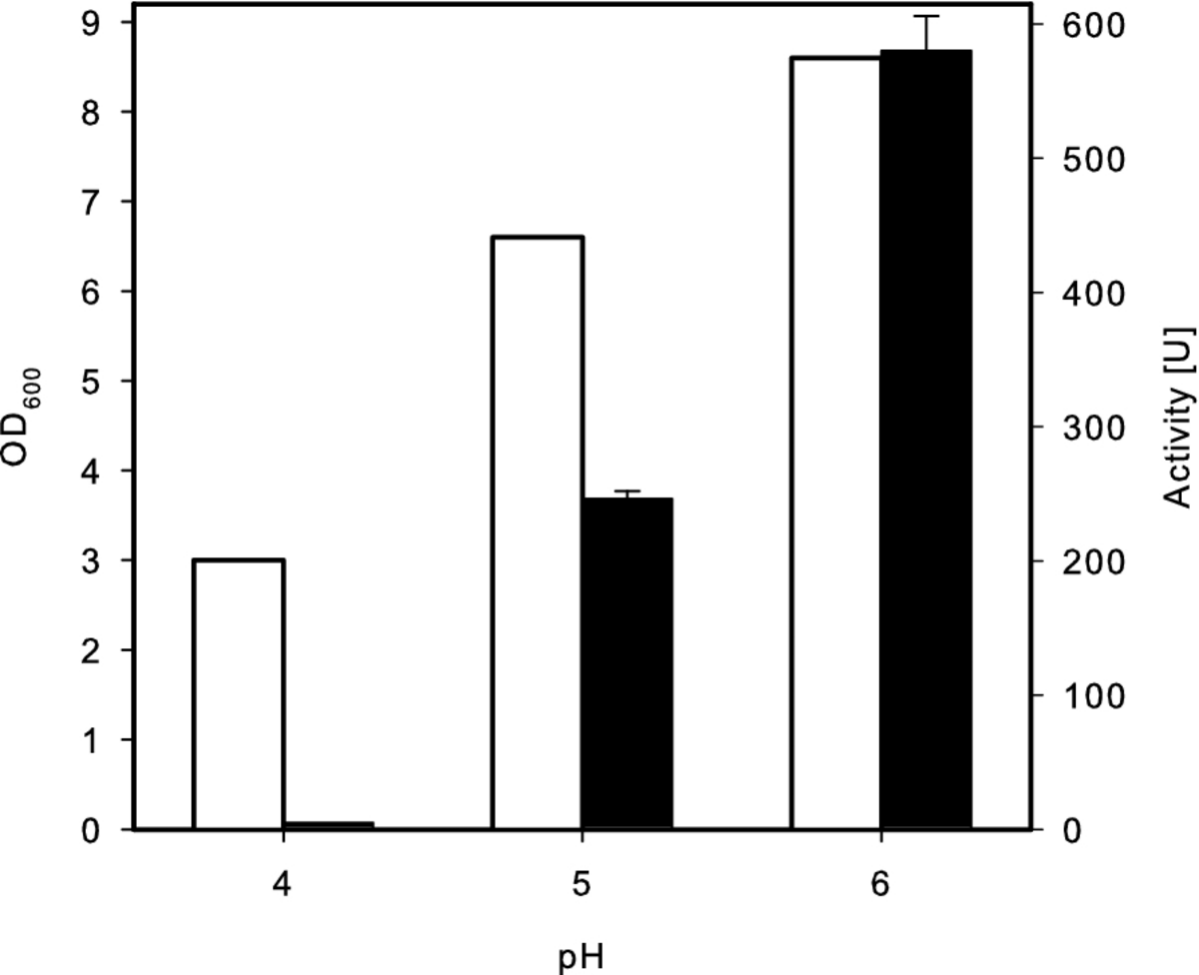
**The optical cell densities (OD_600_) (white bars) and malolactic activities (black bars) at different pH, 24 h after incubation in *L. plantarum *harbouring pSC9mle**. The production of MLE was induced after 5 h of incubation.

Heterologous expression of the MLE in *L. plantarum *was tested at pH 4.0, 5.0 and 6.0. As shown in Figure 3, the OD_600 _was significantly higher after 24 h of cultivation at pH 6.0 and 5.0 compared to pH 4.0. The MLE activity decreased significantly at pH below 6.0 in the medium, although similar protein contents of 13.4 ± 0.5 mg/ml were determined after disruption of equivalent cell quantities. Interestingly, the activity of MLE obtained from the cultivation at pH 5.0 was reduced more than 50% compared to pH 6.0, while the OD_600 _was reduced approximately 25%.

Analysis of the malolactic fermentation in MRS medium at pH 4.0 showed similar cell growth and decarboxylation of L-malic acid of the wild-type and induced recombinant *L. plantarum *strain (data not shown). Both wild-type and the recombinant strain converted 5 g/l L-malic acid in 24 h and the final content of L-malic acid in the medium was found to be 0.025 ± 0.001 g/l (data not shown).

### Conversion of L-malic acid in malolactic test solution and modified wine

To investigate the extent of L-malic acid conversion of the wild-type and the recombinant *L. plantarum *strain, both strains were incubated in a solution containing L-malic acid as well as in a modified white wine. Since the results shown above indicated that the initial pH affects the expression, cells were grown and induced in medium at pH 6.0 before the malic acid conversion experiments were conducted.

**Figure 4:**
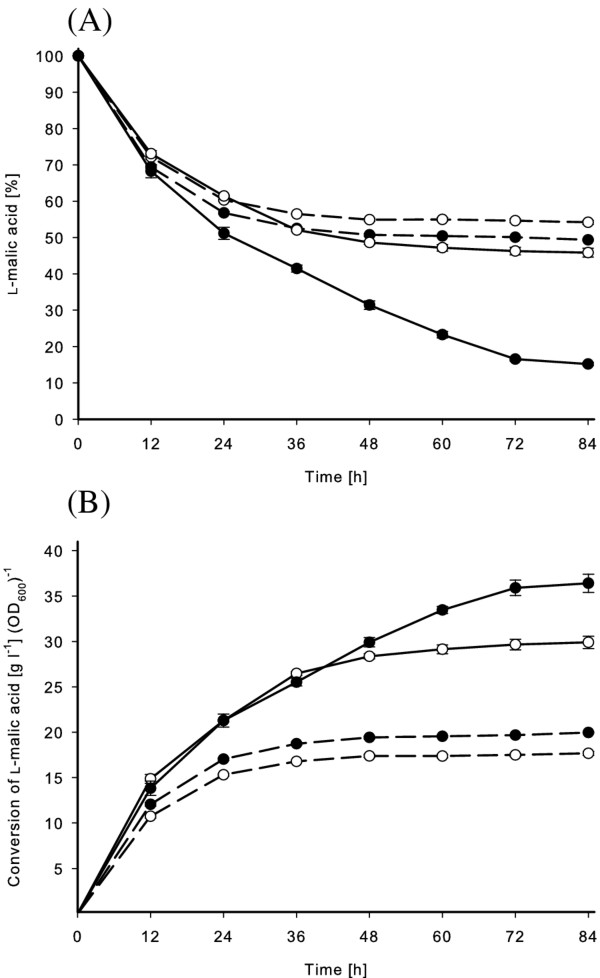
**Conversion of L-malic acid in malolactic test solution**. The wild-type (dashed line) and recombinant *L. plantarum *(solid line) strain were incubated at 25°C in solution containing 3.0 g/L (100%) L-malic acid (pH 5.0). The conversion is displayed without addition (○) and with addition of 0.1 mM NAD^+ ^and 0.02 mM Mn^2+ ^(●). **(A) **shows the time course of malic acid conversion and **(B) **presents the conversion of L-malic acid in relation to the cell density. All experiments were carried out in triplicate.

The cells were harvested after 8 h and subsequently diluted to OD_600 _of ~0.1 (2.8 × 10^9 ^CFU/ml) for both strains to be used for L-malic acid conversion analysis. No detectable intracellular MLE activity was observed for the wild-type strain, on the other hand, 0.2 U/ml was determined for the recombinant cells used for the conversion of L-malic acid. The recombinant strain continuously converted L-malic acid reaching up to 85% (2.5 g/l) in 3.5 days when the cofactors Mn^2+ ^and NAD^+ ^were present (Figure 4A). Figure 4B shows the conversion rates in relation to the cell density, indicating that the recombinant strain showed enhanced conversion, even in absence of the cofactors. In cultures with 0.02 mM Mn^2+ ^and 0.1 mM NAD^+^, L-malic acid conversion increased by 13% and 22% of the wild-type and the recombinant *L. plantarum *after 84 h, respectively.

**Figure 5:**
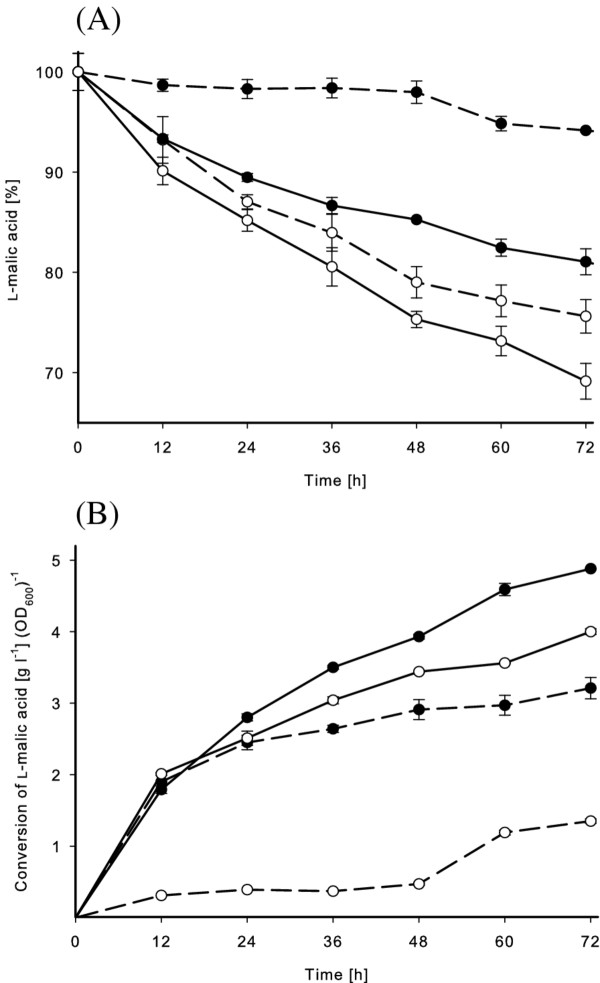
**Bioconversion of L-malic acid in modified wine in a discontinuous batch process**. The fermentations of wild-type (dashed line) and recombinant *L. plantarum *(solid line) were carried out at 25°C in modified wine. The initial L-malic acid concentration (each calculated as 100%) for fermentation using pre-adapted cells to wine (○) and for non adapted cells (●) was 3.3 g/l (pH 5.0) and 2.9 g/l (pH 5.2), respectively, containing Mn^2+ ^(0.02 mM) and NAD^+ ^(0.1 mM). **(A) **shows the time course of L-malic acid conversion and **(B) **presents the conversion of L-malic acid in relation to the cell density. All experiments were carried out in triplicate with standard deviation shown as error bars.

The conversion of L-malic acid was also tested using modified wine in a discontinuous fermentor. The application of the cells showed that the non-adapted wild-type *L. plantarum *hardly converted L-malic acid at all during the analysis period, while adapted cells continuously converted L-malic acid (Figure 5A). The non adapted recombinant cells converted 15% (0.4 g/l) L-malic acid in 48 hours. The adapted recombinant cells converted even 25% of inital L-malic acid, although pre-adaption to wine resulting in 5 times less intracellular activity (data not shown). Furthermore, pre-adapted cells increased their density during incubation in modified wine. Taking the different cells densities into account, Figure 5B shows that non adapted recombinant cells showed highest conversion rate.

When using the recombinant *L. plantarum *strain, which was cultivated and induced in the absence of L-malic acid, no decarboxylation was observed from the malolactic test solution (data not shown).

## Discussion

At present, genetically modified organisms are already used in winemaking. Recombinant *Saccharomyces *strains conduct simultaneous alcoholic and malolactic fermentation, however, if the distinct aroma produced during MLF is desired, the use of LAB is necessary. Although *O. oeni *is the preferred microorganism to conduct MLF, recent research showed that *L. plantarum *is a promising candidate to reduce L-malic acid in wine as well (Pozo-Bayon et al. 2005, du Toit et al. 2010). Not only that *L. plantarum *occurs naturally at different stages during wine making, it is also of interest as efficient antimicrobial agent to control spoilage microorganisms in winemaking (Navarro et al. 2008). Furthermore, wine related *Lactobacillus *species, including *L. plantarum*, are as efficient as *O. oeni *with excellent potential as starter cultures (du Toit et al. 2010). In this study we present a *L. plantarum *strain which produces recombinant MLE from *O. oeni *for enhanced MLF. The induced recombinant strain converted more L-malic acid and adapted better to wine condition than the wild-type *L. plantarum*.

This is the first report on the successful heterologous expression of MLE in a *Lactobacillus *spp. The *L. plantarum *WCFS1 which is a model strain in LAB research was selected for this study. The pSIP expression system (Sørvig et al. 2003) is well thoroughly tested in this strain and may produce level of the target protein to more than the half of total cellular proteins (Halbmayr et al. 2008). Previous attempts to conduct the MLF by recombinant *Lactobacillus *failed due to shuttle vector instability (Chagnaud et al. 1992). Although, the expression in *L. plantarum *strain (this study) is not as efficient as in *E. coli*, the enzyme activity obtained was 10 times higher compared to the native *O. oeni *strain (Schümann, personal communication). By comparing the specific activities of the crude MLE from *L. plantarum *with different expression hosts, the activity obtained in the present study is clearly the highest detected so far (Table 1). This illustrates the usefulness of the pSIP system for efficient production of malolactic enzyme in *L. plantarum*.

In the present study, we wanted to determine the ability of the recombinant *L. plantarum *strain to increase MLF compared to the wild-type strain. The application of MLF to wine necessitates the adaptation of microorganism to low pH, therefore it is important to consider the influence of acidity on the used expression system. Our results showed that the expression of the recombinant strain was highest in a medium at pH 6.0 and induction in the presence of even diluted wine decreased the expression level. Expression in a medium at pH 4.0 led to undetectable activity and the application in MRS medium (pH 4.0) resulted in similar conversion of the added L-malic acid (5 g/l) compared to the wild-type strain. This is probably at least partly due to loss of the expression plasmids from the *L. plantarum *at low pH. It has previously been shown that the erythromycin gene which is used as selection for the plasmid has minimal activity at low pH (Lorian and Sabath 1970), and therefore the bacteria will dispose of the plasmid. To overcome this problem, *L. plantarum *cells were cultured and induced in a medium at pH 6.0, where cells showed highest malolactic activity, and then harvested and directly inoculated to L-malic acid containing solutions with pH 5.0-5.2. Using already induced cells as starter cultures enabled the application in the absence of the antibiotics and the inducing agent. The increased intracellular MLE activity probably enabled the recombinant strain to convert L-malic acid more efficiently than the wild-type strain and furthermore to survive longer under harsh conditions with no other substrate rather than L-malic acid (Figure 4B).

The role of Mn^2+ ^and NAD^+ ^is not exactly clear due to similar rates of malic acid conversion during the first 36 h of reaction time (Figure 4A). It might be that the intracellular cofactors were metabolized to yield energy and subsequently to ensure survival of the cells.

The induced recombinant cells, pre-cultured without L-malic acid, were not able to convert L-malic acid in malolactic test solution, although significant intracellular MLE activity was detected. An explanation might be the lack of malate permease, responsible for the transport of malic acid into the cell, which is only expressed in presence of malic acid (Bandell et al. 1997).

The application of *L. plantarum *to modified wine showed that pre-adaption was necessary for the wild-type strain, while the recombinant strain was able to directly convert L-malic acid (Figure 5B). Interestingly, adapted recombinant cells, on the other hand, had a lower conversion rate per cells than non adapted cells. The reason might be either due to the inhibited expression during the cultivation in the presence of wine or the absence of Mn^2+ ^and NAD^+^.

The advantage of over-expressing the MLE in *L. plantarum *for MLF was demonstrated in the present study. The pSIP expression system and the 'model' strain *L. plantarum *WCFS1 used in this study demonstrated that the expression of MLE in *Lactobacillus *is feasible and improved malolactic fermentation can be achieved. However, some optimizations have to be done for application in wine. The antibiotic resistance marker needs to be exchanged to a food-grade selection marker. This has recently been done with the pSIP vectors using *alr *gene as plasmid selection marker instead of the erythromycin gene. This system has successfully been applied for overexpression of a β-galactosidase (Nguyen et al. 2011).

In summary, this study showed the advantage of the recombinant *L. plantarum*, heterologously expressing the MLE, in terms of more efficient degradation of L-malic acid and better adaption to wine conditions compared to the wild-type strain.

## Competing interests

The authors declare that they have no competing interests.
